# Effects of local cardiac denervation on cardiac innervation and ventricular arrhythmia after chronic myocardial infarction

**DOI:** 10.1371/journal.pone.0181322

**Published:** 2017-07-21

**Authors:** Xudong Liu, Lin Sun, Jugang Chen, Yingying Jin, Qing Liu, Zhongnan Xia, Liang Wang, Jingjie Li

**Affiliations:** 1 Department of Cardiology, The First Affiliated Hospital of Harbin Medical University, Heilongjiang Province, Harbin city, PR China; 2 Department of Cardiology, The First Affiliated Hospital of Xingxiang Medical University, Henan Province, Xinxiang city, PR China; University of Minnesota, UNITED STATES

## Abstract

**Background:**

Modulation of the autonomic nervous system (ANS) has already been demonstrated to display antiarrhythmic effects in patients and animals with MI. In this study, we investigated whether local cardiac denervation has any beneficial effects on ventricular electrical stability and cardiac function in the chronic phase of MI.

**Methods:**

Twenty-one anesthetized dogs were randomly assigned into the sham-operated, MI and MI-ablation groups, respectively. Four weeks after local cardiac denervation, LSG stimulation was used to induce VPCs and VAs. The ventricular fibrillation threshold (VFT) and the incidence of inducible VPCs were measured with electrophysiological protocol. Cardiac innervation was determined with immunohistochemical staining of growth associated protein-43 (GAP43) and tyrosine hydroxylase (TH). The global cardiac and regional ventricular function was evaluated with doppler echocardiography in this study.

**Results:**

Four weeks after operation, the incidence of inducible VPC and VF in MI-ablation group were significantly reduced compared to the MI dogs (*p<*0.05). Moreover, local cardiac denervation significantly improved VFT in the infarcted border zone (*p*<0.05). The densities of GAP43 and TH-positive nerve fibers in the infarcted border zone in the MI-ablation group were lower than those in the MI group (*p*<0.05). However, the local cardiac denervation did not significantly improve cardiac function in the chronic phase of MI, determined by the left ventricle diameter (LV), left atrial diameter (LA), ejection fraction (EF).

**Conclusions:**

Summarily, in the chronic phase of MI, local cardiac denervation reduces the ventricular electrical instability, and attenuates spatial heterogeneity of sympathetic nerve reconstruction. Our study suggests that this methodology might decrease malignant ventricular arrhythmia in chronic MI, and has a great potential for clinical application.

## Introduction

Modulation of the sympathetic nervous system is an effective method to reduce cardiac arrhythmia in patients and animals suffering from myocardial infarction (MI). Many procedures, such as beta-blocker application[1–3], cardiac sympathetic denervation, thoracic epidural anesthesia and renal sympathetic denervation etc[4–7], can reduce the activity of the sympathetic nervous system. However, these regimens block sympathetic nerve fibers entering not only cardiac tissue but also other organs, causing the dysfunction of other organs and unwanted complications. To avoid these potential side effects, we developed the local cardiac denervation, in which the coronary sinus (CS) and great cardiac vein (GCV) peripheral nerves are ablated in situ. The local cardiac denervation reduces ventricular arrhythmias (VA) in a canine acute MI model[8], suggesting it may be a novel methodology to prevent VA complicating acute MI.

Neural remodeling and ventricular electrical instability contribute to fatal arrhythmia, especially in the chronic phase of MI [9]. Whether the local cardiac denevation has any beneficial effects on ventricular electrical stability and cardiac function in the chronic phase of MI are largely unknown. In this study, with the canine MI model, we demonstrated that the local ablation of CS and GCV peripheral nerves reduced the induction rate of VA at the chronic phase of MI, inhibited cardiac nerve sprouting and improved ventricular electrophysiology. This regimen did not display obvious side effects in terms of heart rate, systemic arterial pressure, infarct size and cardiac function.

## Materials and methods

### Experimental animals

Mongrel dogs of both sexes were specifically bred by the experimental animal center at the First Affiliated Hospital, Harbin Medical University, China. All experimental procedures involving animals were conducted as per the Institutional Animal Care guidelines and were ethically approved by the Administration Committee for Experimental Animals Heilongjiang Province China. The experimental animals were under light and dark cycles of 12 hours, room temperature ranging from 20–25 uC, and water and chow ad libitum. Every two dogs were kept in 4 m^2^ in the kennel. Twenty-one dogs, weighing between 15 and 25 kg, were anesthetized with sodium pentobarbital (25 mg/kg induction; 1.0 mg/kg/h with intermittent boluses, as needed), and then intubated and mechanically ventilated (Electrical Animal Ventilator, Medical Equipment Factory, Shanghai, China) to maintain the arterial pCO2 between 35 and 40 mm Hg. Fluid resuscitation was established with 0.9 N NaCl at 10 ml/kg/h. The systemic arterial pressure was monitored during the experiment with a computer-based Lab System (GY-6328, Huanan Inc., China). The core body temperature of the animals was maintained at 36.5 ± 1.5°C with heating pads.

### Generation of myocardial infarction and local ablation of cardiac sympathetic nerves

Twenty one animals were randomly assigned into the groups of sham-operated (n = 5), myocardial infarction (MI, n = 8) and myocardial infarction followed by nerve ablation (MI-ablation, n = 8). To establish MI in both the MI and the MI-ablation groups, the left anterior descending coronary artery (LAD) was permanently ligated just below the first diagonal branch by the one-stage procedure in the open-chest and anesthetized dogs. The animals in the sham-operated group also underwent thoracotomy and pericardiotomy, but not coronary artery ligation[10].

**Fig 1:**
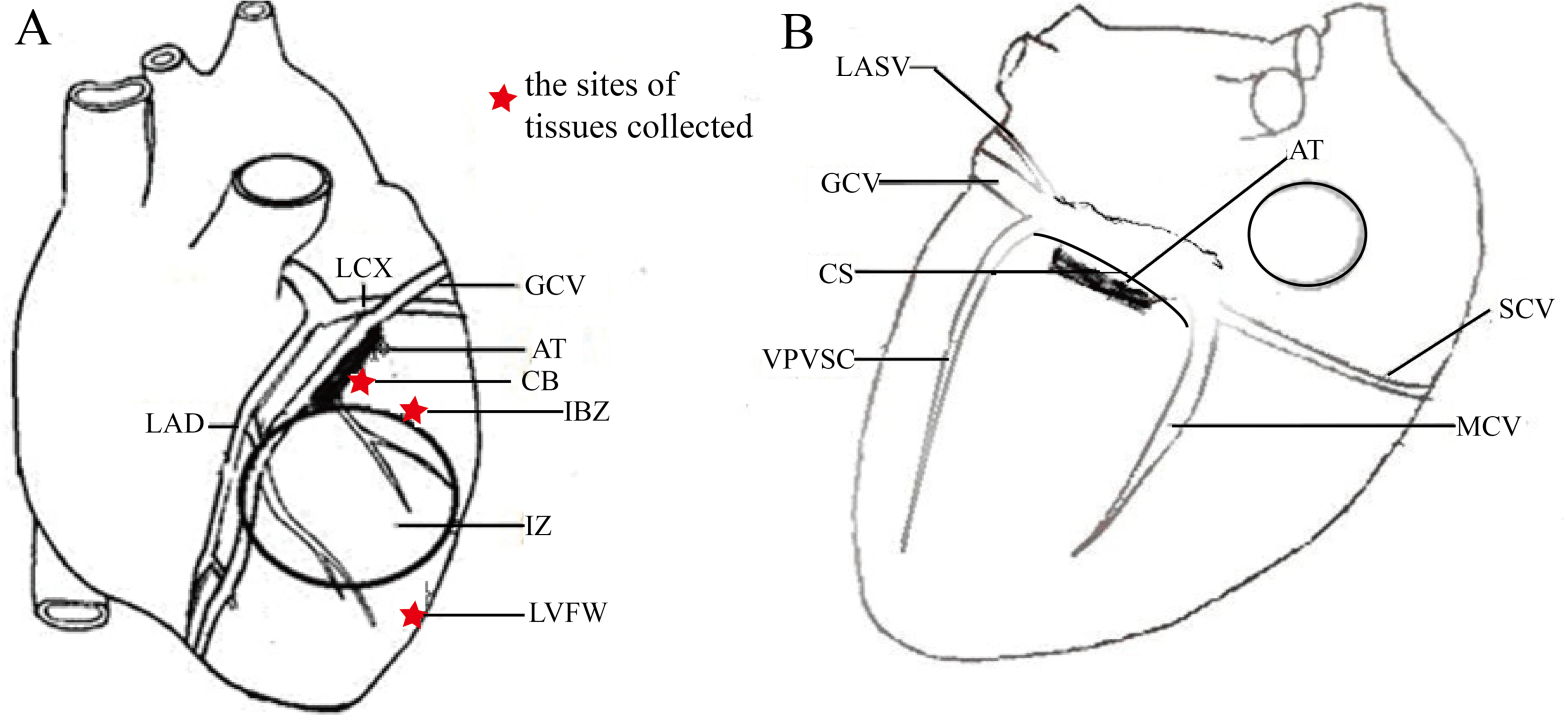
**A sketch of the anterior (A) and posterior (B) of a dog heart**, in which the ablation targets, the multi-electrodes, the infarct area, the coronary artery and Coronary Sinus and its tributaries are shown. AT, ablation target; IZ, infarction area; LAD, left anterior descending coronary artery; LCX, left circumflex coronary artery; CS, coronary sinus; GCV, great cardiac vein; MCV, middle cardiac vein; SCV, small cardiac vein; LASV, left atrium slanting veins; VPVSC, venae posterior ventriculi sinistri cordis.

After successfully creating MI, we gave the animals a 90-minute interval to recover. The dogs in the MI-ablation group then further underwent cardiac nerves ablation. The ablation sites were determined by the anatomical distribution of autonomic nerves along the CS (the distal CS, proximal 2 cm from the coronary sinus ostium) and the GCV (upper third anterior interventricular groove) (Fig 1). To avoid a potential damage of the proximal LAD and left circumflex (LCX) arteries, which distribute in the target area, the ablation sites were also chosen 0.5cm away from these arteries. The radiofrequency (RF) energy of a cardiac ablation generator (IBI—1500T8, Irvine Biomedical Inc., USA) was set 20 W with a cut-off temperature at 60°C, and a saline irrigation catheter (7-F, 4.0-mm tip electrode, Irvine Biomedical Inc. USA) was applied to ablate the nerves for 2 min at both sides of the CS and GCV, respectively. The ablation was performed in the epicardium of the MI-ablation group. An open irrigation system was set to continuously irrigate the ablation catheter at 40–60 mL/min during radiofrequency delivery.

After surgery, the thorax was closed, and pleural air was aspirated. The dogs were allowed to recover, and received analgesia and antibiotic therapy by Tramadol (0.02 ml/kg induction as needed). Dressing and drainage in the first 4 days. Daily observation of the general state, monitoring of vital signs. No extra exercises was provided to the dogs. Four weeks later, two dog in MI group was dead, other survived animals were returned to the laboratory for further experimental study.

### Evaluation of cardiac function

Four weeks after ablation or the generation of MI, the cardiac function of all survival dogs was evaluated. The left ventricle diameter (LV), left atrial diameter (LA) and ejection fraction (EF) were determined with doppler echocardiography. Briefly, the left atrial anteroposterior dimension was measured during LV end-systole immediately before mitral valve opening from the parasternal long-axis view. LV end-diastolic dimension, inter-ventricular septal thickness, and posterior wall thickness were measured during LV end-diastole immediately before aortic valve opening from the parasternal long-axis view. The LV ejection fraction (LVEF), the index of global LV systolic function, was computed from apical two-and four-chamber views. LVEF was measured using the biplane modified Simpson method to assess LV global function.

### Electrophysiological study

After the evaluation of cardiac function, dogs were anesthetized with sodium pentobarbital again and other experimental conditions as 4 weeks before. The left stellate ganglion was dissected and isolated from surrounding connective tissue at the first and second intercostal space of left thoracic. Then central connections were cut at junction of ansae subclaviae and stellate ganglion, and a shielded platinum stimulating electrode was placed firmly around proximal end. Stellate stimulation consisted of repeated square wave pulses (5- min duration and 10-min interval) delivered at 10 to15 Hz with stimulus amplitude of 10 V. Stimulation was effective when stimulated to increase mean arterial pressure by more than 20 mm Hg. The continuous ECG was recorded in all dogs for 1h during stellate stimulation to determine the incidence and duration of VA, including ventricular premature contraction (VPC), paroxysmal ventricular tachycardia (PVT) and spontaneous VF[11–12]. If a sustained VF was induced, the stellate stimulation was termination and a cardiac electric defibrillator was used to shock the heart back to normal rhythm.

The VFT, the minimum voltage to induce sustained VF, was determined in the all survival dogs. Thirty minutes after left stellate ganglion stimulation, VF was induced at the same heart rate (200 beats/min) among animals. Followed by a 20-beat drive train with a pacing cycle length of 300ms, 100ms S1–S1 stimuli were repeatedly applied to the right ventricular apex with an increase of stimuli intensity by 2V each time until VF was induced. Each stimulus lasted for 10 second and followed by a 30-second rest period before the next round of stimulation. Once a sustained VF was induced, a cardiac electric defibrillator was used to shock the heart back to normal rhythm. After a 5-minute break, the stimulation protocol was repeated to measure the second VFT. The measurements of both times were averaged as mean VFT. At the end of experiment, all dogs died of sustained VF. Animal deaths were confirmed by observation of dog respiratory and cardiac arrest.

### Histology immunohistochemistry

At the end of the experiment, the hearts were quickly collected from all dogs in each group. (S1 Fig) These sites were equally split into three groups which distributed respectively in the infarcted border zone (IBZ between infarcted zone and the noninfarcted left ventricle free wall, within 3mm in the pale marginal area of infarcted zone), the noninfarcted left ventricle free wall (LVFW, beyond 2cm in the pale marginal area of infarcted zone) and cardiac base (CB, the ablation sites of around GCV). The size of about 0.6 × 0.8 cm. 4 μm transmural sections were cut perpendicularly to the epicardium and mounted onto slides. Immunohistological staining of growth associated protein-43 (GAP43) and tyrosine hydroxylase (TH) was carried out according to standard procedures. The density of TH-positive sympathetic nerve fibers was quantified using Motic Images Advanced 3.2 software and expressed as the nerve area divided by the total area examined (μm2/mm2). The nerve density of each slide was determined by the average of 3 areas with the highest nerve density.

### Measurement of infarct size

Triphenyl tetrazolium chloride (TTC) was used to distinguish infarct area from noninfarcted tissue according to dye-exclusion method described previously [13]. The unstained infarct tissue was mechanically separated from the brick red-stained noninfarcted left ventricular wall. The mass of the infarct tissue was weighed and normalized with the left ventricular tissue mass.

### Statistical analysis

The measurement data were expressed as mean ± standard deviation (± s). The Student-Newman- Keuls Q test which was used to compare, ventricular fibrillation threshold, TH, GAP43, heart rate, and blood pressure across groups; the Kruskal-Wallis H rank-sum test was used between the three groups of non-normal distributions, the Nemenyi test And duration); Between group differences in myocardial infarction size were compared using the Student t-test. The software SPSS 17.0 (SPSS, Chicago, IL, USA) was used in the statistical analysis. Comparisons among continuous data were performed using a one-way analysis of variance (ANOVA); whereas categorical data were analyzed with the *χ*^2^-test. Values of p *<* 0.05 were considered statistically significant.

## Results

### General results

Sixteen MI animals were generated by ligating the left anterior descending coronary artery. After ligation, the ECG immediately displayed ST-segment elevation, and the lesional epicardium gradually became pale. Eight ligated dogs further underwent the radiofrequency ablation of the cardiac sympathetic nerves along the CS and GCV. The ablation sites, shown in Fig 1A and 1B, were 0.5 cm away from LAD and LCX. During surgery, 3 out of 16 ligated dogs displayed VPC and PVT. Two MI and one MI-ablation animals showed sustained VT. A cardiac electric defibrillator was applied to these animals to recover normal heart rhythm. Although all animals survived surgery, two MI dogs died respectively at the fifth and fourteenth day after surgery unexpectedly. We did open chest autopsy. The former was shown to die of severe chest infection. The latter thoracic cavity and pericardial effusion was obvious and without other obvious signs. We analyzed that death was due to heart failure.

### Electrophysiological study

The local cardiac denervation prevents ventricular arrhythmias (VA) complicating acute MI [8], and thus it could also decrease the probability of VA in the chronic phase of MI. To test this hypothesis, we stimulated the left stellate ganglion to induce artificial VA in all experimental animals, and determined whether the local ablation of cardiac nerves affects VA occurrence. After stimulating the left stellate ganglion, no VF was observed among three groups, but one MI dogs displayed sustained VT. In the MI animals, the stimulation of the left stellate ganglion significantly increased the number of ventricular premature complexes (VPCs), and elevated the episodes and duration of polymorphous ventricular tachycardia (PVT), suggestion ventricullar arrhythmias is very easily induced in chronic MI. However, the local cardiac denervation dramatically decreased VPCs occurrence at the chronic phase of MI after ganglion stimulation (Table 1). Although there is no significant difference, the episodes and durations of PVT were showed a decreased trend in the MI-ablation animals as compared with MI dogs.

**Table 1:** The incidence and duration of ventricular arrhythmias among three groups during one hour of left stellate ganglion stimulation discontinuously($\bar{x}$±s).

	sham-operated group (n = 5)	MI group (n = 6)	MI-ablation Group (n = 8)
VPC	63.0±16.5	249.8±72.6*	102.8±22.1#*
PVT	0	2.0±1.8*	0.4±0.5
durations of PVT (s)	0	3.2±1.7*	0.8±1.1

Note

* *p* < 0.05 vs. sham-operated group

# *p* < 0.05 vs. MI group.

At the end of the electrophysiological study, we determined ventricular fibrillation threshold (VFT) by purposely inducing VF in all live animals. Comparing with the MI group, the local denervation significantly increased the VFT in MI-ablation dogs. Furthermore, the increased VFT in the MI-ablation group had no significant difference from that in the sham-operated group (Table 2).

**Table 2:** The ventricular fibrillation threshold among three groups($\bar{x}$±s).

	sham-operated group (n = 5)	MI group (n = 6)	MI-ablation group (n = 8)
Ventricular fibrillation threshold (V)	19.2±4.1	10.7±2.2*	15.8±2.6#

Note

* *p* < 0.05 vs. sham-operated group

# *p* < 0.05 vs. MI group.

Summarily, above result demonstrated that the local cardiac denervation inhibits VA occurrence in the chronic phase of MI.

### Nerve sprouting and sympathetic hyperinnervation

**Fig 2:**
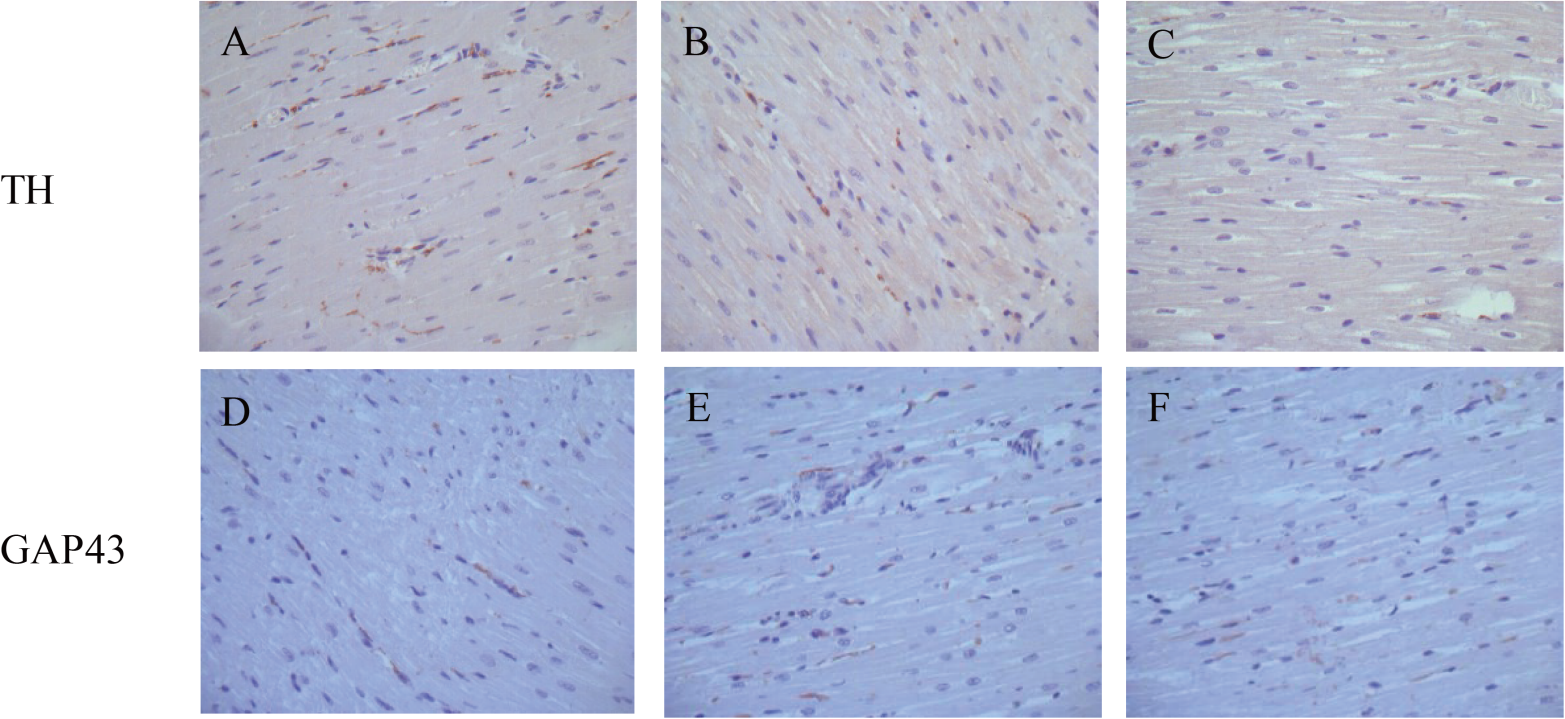
**The TH and GAP43-positive sympathetic nerve fibers in the infarcted border zone (×200) in the sham-operated group (A, D), the MI group (B, E) and MI-ablation group (C, F).** Positive nerve fibers were shown in brown color and located between myofibrils. The TH and GAP43-positive sympathetic nerve fibers in MI-ablation group were decreased when compared with MI group.

**Fig 3:**
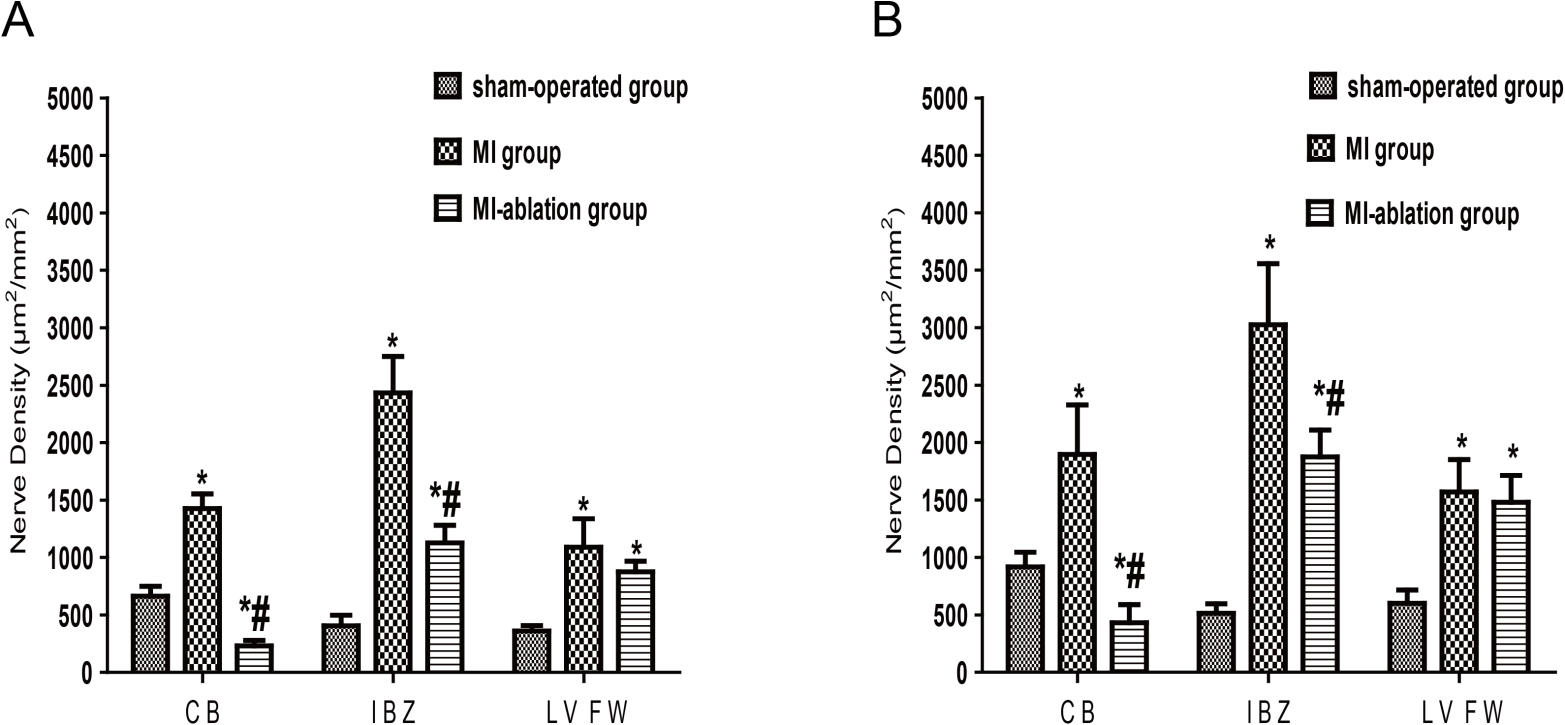
**The density of TH (A) and GAP43 (B) positive nerve fibers around the CB, IBZ andnon-infarcted LVFW in the sham-operated, MI and MI-ablation group.** MI, myocardial infarction; TH, tyrosine hydroxyls; GAP43, growth associated protein 43; CB, LV anterior/ventral base; IBZ, infarction border zone; LVFW, left ventricle free wall. Note: * *p* < 0.05 vs. sham-operated group; # *p* < 0.05 vs. MI group.

The cardiac nerve sprouting and sympathetic hyperinnervation[14] are critical determinants for VA occurrence in the MI[13]. To determine effects of the local cardiac denervation on cardiac nerve sprouting and sympathetic hyperinnervation, we performed immunohistological staining of cardiac tissue (IBZ, LVFW and CB) with antibodies against GAP-43, a marker of nerve sprouting, and TH, a marker of sympathetic nerve. During the recovery from myocardial infarction, cardiac nerve sprouting and sympathetic hyperinnervation significantly enhanced in the infarcted area, manifested with the increased numbers of both GAP-43 and TH positive nerves in the CB, IBZ and LVFW of the MI animals. The radiofrequency ablation of cardiac nerves drastically reversed this anomaly, especially in the areas of CB and IBZ (Fig 2). Quantitative data also showed that the densities of either GAP43- or TH positive nerves in the IBZ and CB were significantly lower *(p*<0.05) in the MI-ablation group than that in the MI group (Fig 3). These results suggest that local cardiac denervation inhibits cardiac nerve sprouting and sympathetic hyperinnervation at the chronic pahse of MI.

### Cardiac function, vital signs and infract size

To observe whether the local cardiac devervation has any beneficial effect on the recovery of cardiac function in the chronic phase of MI. Four weeks after surgery, the cardiac function of all survival dogs was determined with doppler echocardiography. As shown in Table 3, the local cardiac denervation did not significantly improved the cardiac function, determined by the left ventricle diameter (LV), left atrial diameter (LA), ejection fraction (EF) and *fractional shortening* (*FS*).

**Table 3:** The cardiac function among three groups ($\bar{x}$±s).

	LA(mm)	LV(mm)	EF(%)	FS(%)
sham-operated group (n = 5)	35±2.1	40±3.1	68±2.3	34±6.1
MI group (n = 6)	39±3.3*	46±4.4*	43±5.5*	22±3.7*
MI-ablation group (n = 8)	38±3.4	49±2.4	40±7.3	26±5.4

Note

* *p* < 0.05 vs. sham-operated group

The alteration of sympathetic nervous system is usually associated with the changes of heart rate and blood pressure[15–16]. However, the local ablation of cardiac sympathetic nerves did not alter these indices when compared with MI and sham-operated animals (Table 4), suggesting this regimen does not cause the systemic side effects and potential damages to other organs.

**Table 4:** The change of vital signs and infarct size among three different groups ($\bar{x}$±s).

	sham-operated group (n = 5)	MI group (n = 6)	MI-ablation group (n = 8)
heart rate (beat/min)	117.6±14.3	147.7±13.7*	139.5±15.7*
Systolic pressure (mmHg)	121.0±8.1	110.7±10.8	106.6±11.5
diastolic pressure (mmHg)	58.0±8.6	53.0±6.5	50.5±7.8
infracts size (%)	——	33.9±3.0	31.7±1.9

Note

* p < 0.05 vs. sham-operated group

## Discussion

In the present study, we found that the local cardiac denervation decreased VPC burden and increased VF threshold at the chronic phase of MI. The local blockade of cardiac sympathetic nerves led to the amelioration of the ventricular, as well as the reduction of cardiac nerve sprouting and sympathetic hyperinnervation. Furthermore, there was no obvious changes of cardiac function, heart rate, systolic pressure, diastolic pressure and infarct size after local cardiac denervation.

Neural remodelling after myocardial infarction, characterized by heterogeneous cardiac nerve sprouting and sympathetic hyperinnervation in infarcted myocardium, may predispose to spontaneous VF[17–19]. Hyperinnervation in infarcted tissues results into the spatially heterogeneity in sympathetic nerve density and the various levels of sympathetic neurotransmitters, consequently leading to spatial heterogeneous electrical remodeling of cardiomyocytes[20]. These electroanatomic remodeling after MI increases I_Ca.L_ density and decreases K^+^ current densities, resulting in action potential prolongation in hyperinnervated regions. The action potential prolongation and augmented Ca^2+^ influx through L-type Ca^2+^ channels synergistically increase the susceptibility to EAD- and/or DAD-triggered activity in hyperinnervated regions, further increasing vulnerability to VA [21]. The importance of neural remodelling in VA is stressed by the efficacy of β-blocker and angiotensinconverting enzyme inhibitor (ACEI) therapy, which dramatically reduce the sudden death after MI[22–25]. Therefore, intervention of neural remodelling after MI may provide a novel opportunity for VA treatment.

The local cardiac dernervation efficiently prevents VT complicating AMI through improving cardiac electrical stability[8]. Our present study further demonstrated that local cardiac denervation also benefits chronic MI by decreasing VPC burden and increased VF threshold. The local ablation of cardiac sympathetic nerves significantly reduces cardiac nerve sprouting and hyperinnervation in the infarcted area at the chronic phase of MI, leading to stabilization of ventricular electrical activity and reduction of VA occurrence. Since the mechanistic relationship between the sympathetic nervous system and VA are very complicated, the other mechanisms may also contribute the beneficiary effects of the ablation of cardiac sympathetic nerve in the chronic phase of MI.

Our previous and present studies proposed a new possibility that the local cardiac denervation may be promising in clinical practice. Percutaneous coronary intervention (PCI) revascularization procedure combined with local cardiac denervation will not only re-establishes the blood transportation system, but also prevent the VA occurrence at both the acute and chronic phases of MI. This combination therapy may be a feasible way to prevent VA and sudden death in MI patients with high risk of VA, and would dramatically improve the outcomes of these patients. Our previous and present studies also demonstrate the safety of the local cardiac denervation. Other popular methodologies of cardiac denervation at sites far from the heart, including the removal of unilateral stellate ganglion, thoracic epidural anesthesia, renal sympathetic denervation, etc[4–7], blocks sympathetic nerve fibers entering not only cardinal tissue but also other organs, causing the dysfunction of other organs and unwanted complications. Compared with these regimens, the local cardiac denervation does not cause the systemic side effects, and have more bright perspective in clinical application.

Although our studies indicate that the local cardiac denervation may prevent acute and chronic MI-related VA, further studies will be needed to address: (a) the molecular and cellular mechanism of the beneficiary effect of the local cardiac denervation; (b) non-invasive assessment of successful denervation *in vivo*; (c) the difference of experimental infarction in our study versus coronary clinical infarction. (d) Patients with chronic myocardial infarction often suffer from recurrent monomorphic ventricular tachycardias. However, the incidence of recurrent monomorphic ventricular tachycardias in the experiment is low. We have not been able to fully simulate clinical patients by giving a stimulating approach. So we take the stimulus method to increase the monomorphic ventricular tachycardia and ventricular premature frequency. (e) The durability of this regimen is still unclear. This methodology could be temporary as the cell bodies of these fibers remain intact in the stellate and other thoracic ganglia.

All the primary data were shown in S1–S7 Tables.

## Supporting information

S1 Fig The MI seen in epicardium after second thoracotomy. (JPG)

S1 Table The primary data of GAP43 positive nerves. (XLS)

S2 Table The primary data of TH positive nerves. (XLS)

S3 Table The threshold of ventricular fibrillation. (XLS)

S4 Table The occurrence of ventricular arrhythmia. (XLS)

S5 Table The area of ischemic myocardium. (XLS)

S6 Table The heart function of each dog. (XLS)

S7 Table The heart rate and blood pressure of each dog. (XLS)
