# Supplementary figures and images for: Effects of local cardiac denervation on cardiac innervation and ventricular arrhythmia after chronic myocardial infarction
Source: PLoS One. 2017 Jul 21;12(7):e0181322. doi: 10.1371/journal.pone.0181322 (PMC5521775; doi:10.1371/journal.pone.0181322)

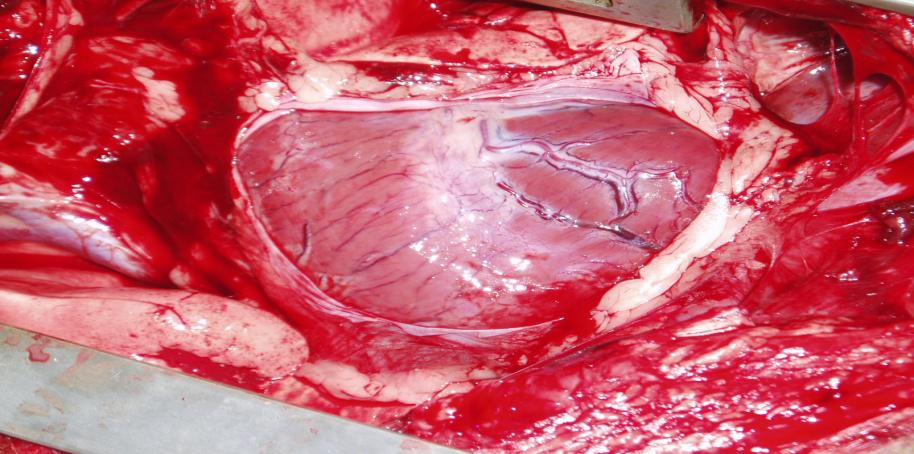

Supplement: S1 Fig — (JPG) [file pone.0181322.s001.jpg]
